# Phylogenomic characterization and signs of microevolution in the 2022 multi-country outbreak of monkeypox virus

**DOI:** 10.1038/s41591-022-01907-y

**Published:** 2022-06-24

**Authors:** Joana Isidro, Vítor Borges, Miguel Pinto, Daniel Sobral, João Dourado Santos, Alexandra Nunes, Verónica Mixão, Rita Ferreira, Daniela Santos, Silvia Duarte, Luís Vieira, Maria José Borrego, Sofia Núncio, Isabel Lopes de Carvalho, Ana Pelerito, Rita Cordeiro, João Paulo Gomes

**Affiliations:** 1 Genomics and Bioinformatics Unit, Department of Infectious Diseases, National Institute of Health Doutor Ricardo Jorge (INSA), Lisbon, Portugal; 2 Technology and Innovation Unit, Department of Human Genetics, National Institute of Health Doutor Ricardo Jorge (INSA), Lisbon, Portugal; 3 National Reference Laboratory of Sexually Transmitted Infections, Department of Infectious Diseases, National Institute of Health Doutor Ricardo Jorge (INSA), Lisbon, Portugal; 4 Emergency Response and Biopreparedness Unit, Department of Infectious Diseases, National Institute of Health Doutor Ricardo Jorge (INSA), Lisbon, Portugal; 5 Faculty of Veterinary Medicine, Lusófona University, Lisbon, Portugal

**Keywords:** Viral infection, Viral genetics

## Abstract

The largest monkeypox virus (MPXV) outbreak described so far in non-endemic countries was identified in May 2022 (refs. ^1–6^). In this study, shotgun metagenomics allowed the rapid reconstruction and phylogenomic characterization of the first MPXV outbreak genome sequences, showing that this MPXV belongs to clade 3 and that the outbreak most likely has a single origin. Although 2022 MPXV (lineage B.1) clustered with 2018–2019 cases linked to an endemic country, it segregates in a divergent phylogenetic branch, likely reflecting continuous accelerated evolution. An in-depth mutational analysis suggests the action of host APOBEC3 in viral evolution as well as signs of potential MPXV human adaptation in ongoing microevolution. Our findings also indicate that genome sequencing may provide resolution to track the spread and transmission of this presumably slow-evolving double-stranded DNA virus.

## Abstract (web summary)

Analysis of the first sequences from the 2022 multi-country outbreak of monkeypox virus shows relatedness and substantial divergence from a 2018–2019 outbreak, suggesting rapid virus evolution with possible implications for human-to-human transmission.

## Main

Monkeypox is a rare zoonotic disease that is caused by the MPXV from the Orthopoxvirus genus, which includes the variola virus, the causative agent of smallpox^1–3^. With an incubation period of 5–21 days, human disease typically begins with fever, myalgia, fatigue and headache, often followed by maculopapular rash at the site of primary infection that can spread to other parts of the body^1^. Although the natural reservoir of MPXV remains unknown, animals such as rodents and non-human primates may harbor the virus, leading to occasional spill-over events to humans^1–3^. MPXV is endemic in West and Central African countries, and the rare reports outside these regions have been associated with imports from those endemic countries^1–4^. We are now facing the first multi-country outbreak without known epidemiological links to West or Central Africa^1^, with more than 2,500 confirmed cases reported worldwide as of 18 June 2022 (refs. ^5,6^), since the first confirmed case on 7 May 2022 in the United Kingdom^4^. Several measures are being recommended by international health authorities to contain MPXV transmission^1^, including the use of vaccines for selected close contacts of patients with monkeypox (post-exposure) and for groups at risk of occupational exposure to monkeypox (pre-exposure)^7^. The virus can be transmitted from human to human by close contact with lesions, body fluids, respiratory droplets and contaminated materials^1,3^, but the current epidemiological context poses some degree of uncertainty about the viral transmission dynamics and outbreak magnitude.

International sequencing efforts immediately began to characterize the outbreak-causing MPXV to identify its origin and track its dissemination. Genome data will also inform about the virus evolutionary trajectory, genetic diversity and phenotypic characteristics with relevance for guiding diagnostics, prophylaxis and research. Here we report the rapid application of high-throughput shotgun metagenomics to reconstruct the first genome sequences of the MPXV associated with the 2022 MPXV outbreak, providing valuable genomic and phylogenetic data on this emerging threat.

**Fig. 1:**
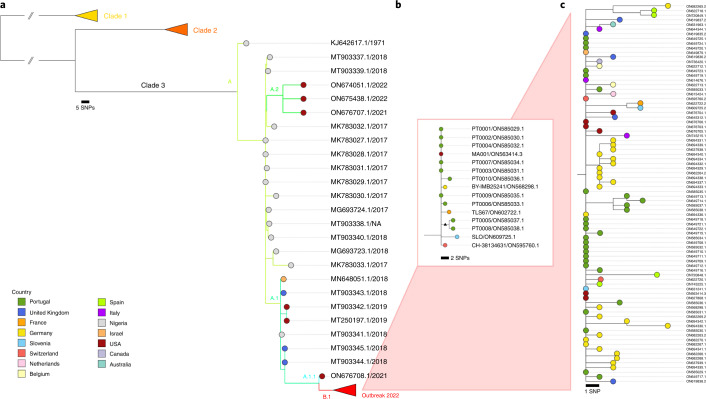
Phylogenetic analysis of MPXV viral sequences associated with the 2022 worldwide outbreak. **a**, MPXV global phylogeny showing that the 2022 outbreak cluster (lineage B.1) belongs to clade 3. Clade and lineage are designated according to the nomenclature proposed by Happi et al.^9^. **b**, Genetic diversity within the outbreak cluster, including the 15 sequences analyzed in this study (released in the NCBI before 27 May 2022). The deletion symbol (Δ) denotes a large deletion (11,335–12,247 in the MPXV-UK_P2-010 gene) shared by sequences segregating in a small subcluster. **c**, Outbreak phylogenetic tree updated with sequences available in the NCBI as of 15 June 2022 (provided during revision for more updated contextualization). The list of the sequences used in these phylogenetic analyses is detailed in Supplementary Table 2, and the alignments are provided as Supplementary Data.

To rapidly get the first insights on phylogenetic placement and evolutionary trends of the 2022 outbreak-causing MPXV, we focused our analysis on a first outbreak-related MPXV genome sequence, publicly released on 20 May 2022 by Portugal^8^, as well as on additional sequences released in the National Center for Biotechnology Information (NCBI) before 27 May 2022, with 15 sequences in total (most of them from Portugal) (Supplementary Tables 1 and 2). The rapid integration of the first sequence into the global MPXV genetic diversity (Fig. 1) confirmed that the 2022 outbreak virus belongs to the MPXV clade 3 (within the formerly designated ‘West African’ clade, which also includes clade 2)^9^. MPXV from clades 2 and 3 are most commonly reported from western Cameroon to Sierra Leone and usually carries a <1% case–fatality ratio (CFR), in contrast with viruses from the clade 1 (formerly designated as ‘Central African’ or ‘Congo Basin’ clade)^9^, which are considered more virulent with a >10% CFR^10,11^. All outbreak MPXV strains sequenced so far tightly cluster together (Fig. 1), suggesting that the ongoing outbreak has a single origin. The 2022 outbreak cluster (lineage B.1)^9^ forms a divergent branch descendant from a branch with viruses (lineage A.1)^9^ associated with the exportation of MPXV in 2018 and 2019 from an endemic country (Nigeria) to the United Kingdom, Israel and Singapore^12,13^, with genetic linkage to a large outbreak occurring in Nigeria in 2017–2018 (ref. ^13^) (Fig. 1). Given these findings and the MPXV historical epidemiology (rare cases in non-endemic countries), it is likely that the emergence of the 2022 outbreak resulted from importation(s) of this MPXV from an endemic country, with the MPXV detected in 2022 potentially representing the continuous circulation and evolution of the virus that caused the 2017–2018 Nigeria outbreak. The recent release of an MPXV sequence from a 2021 travel-associated case from Nigeria to the United States (USA_2021_MD; accession no. ON676708)^14^ phylogenetically placed between 2018–2019 and 2022 sequences (Fig. 1) is aligned with such hypothesis. We cannot, however, exclude the hypothesis of a prolonged period of cryptic dissemination in humans or animals in a non-endemic country (for example, after the reported 2018–2019 importations). Silent human-to-human transmission (for example, due to underdiagnosis) seems less likely considering the known disease characteristics of the affected individuals, usually involving localized or generalized skin lesions^1^. Cryptic transmission in an animal host in a non-endemic country followed by a recent spill-over event is another hypothesis, even though, again, this would be somehow surprising as such a scenario has never been reported. Altogether, current data points for a scenario of more than one introduction from a single origin, with superspreader event(s) (for example, saunas used for sexual encounters) and travel abroad likely triggering the rapid worldwide dissemination^15,16^. Considering the expected incubation period of 5–21 days^3^, limited sampling (including limited viral genotyping data for the first confirmed cases in 2022) and the fact that multiple cases were confirmed in several countries in a 3-week period^1^ after a first report on 7 May 2022 by the United Kingdom^1^, the identification of the index cases associated with such presumable several introductions can be challenging. For example, although the first confirmed case has been hypothesized as the index of the outbreak (due to travel from Nigeria to the United Kingdom on 3–4 May 2022 (refs. ^1,3^)), this scenario can be discarded as the earliest symptom onset dates for confirmed cases in Portugal and in the United Kingdom were in late April^15,16^.

**Fig. 2:**
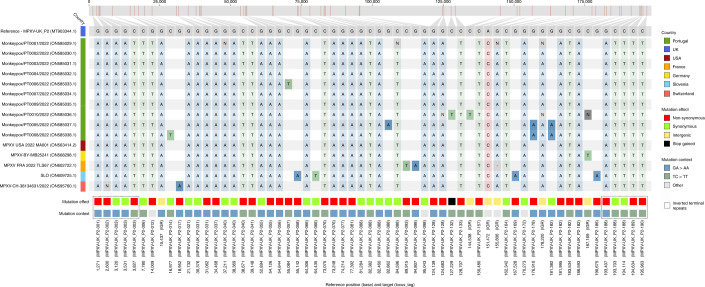
SNPs characterizing the 2022 MPXV outbreak variant. Light-colored mutations represent the SNPs separating the MPXV 2022 outbreak cluster from the MPXV_UK_P2 (MT903344.1) reference sequence (Supplementary Table 3). Dark-colored mutations represent the genetic diversity within the outbreak cluster (Supplementary Table 4).

Notably, the 2022 MPXV diverges from the related 2018–2019 viruses by a mean of 50 single-nucleotide polymorphisms (SNPs) (Figs. 1 and 2), which is far more (roughly 6–12-fold more) than one would expect considering previous estimates of the substitution rate for Orthopoxviruses (1–2 substitutions per genome per year)^17^. Such a divergent branch might represent accelerated evolution. Of note, among the 46 SNPs (24 non-synonymous, 18 synonymous and four intergenic) (Supplementary Table 3) separating the 2022 MPXV outbreak virus from the reference sequence (MPXV-UK_P2, 2018; GenBank accession no. MT903344.1), three amino acid changes (D209N, P722S and M1741I) occurred in the immunogenic surface glycoprotein B21 (MPXV-UK_P2-182)^18^. Serological studies have previously indicated that the monkeypox B21 protein might be an important antibody target with several key immunodominant epitopes^18^. As discussed previously^19^, fine inspection of the mutation profile of those 46 SNPs further revealed a strong mutational bias, with 26 (14 non-synonymous, ten synonymous and two intergenic) and 15 (nine non-synonymous and 16 synonymous) being GA > AA and TC > TT nucleotide replacements, respectively (Fig. 2 and Supplementary Table 3). A tool (https://github.com/insapathogenomics/mutation_profile) was built to rapidly screen these and other mutation profiles. The observed (hyper)mutation signature might suggest the potential action of apolipoprotein B mRNA-editing catalytic polypeptide-like 3 (APOBEC3) enzymes in the viral genome editing^20^. Also, MPXV is A:T rich, so a mutation bias leading to further incorporation of A/T suggests the action of a non-random mutational driver, such as APOBEC3. In fact, APOBEC3 enzymes can be upregulated in response to viral infection, being capable of inhibiting a wide range of viruses by introducing mutations through deaminase and deaminase-independent mechanisms^20,21^. In some circumstances (for example, lower levels of deamination), APOBEC3-mediating mutations might not completely disrupt the virus, thus increasing the likelihood of producing hyper-mutated (but viable) variants with altered characteristics (for example, HIV immune escape variants)^20,22^. The repertoire and level of APOBEC3 enzymes depend on the host species/tissue, and different enzymes display different preferences for the nucleotide or motif (such as dinucleotides or tetranucleotides) to be mutated^20,23,24^. For instance, the GA > AA and TC > TT nucleotide replacements observed in the 2022 outbreak MPXV were also found to be the preferred mutational pattern of human APOBEC3A enzymes (expressed in keratinocytes and skin) during genetic editing of human papillomavirus (HPV) in HPV1a plantar warts and HPV16 pre-cancerous cervical biopsies^25^. Whether the excess of mutations seen in the 2022 MPXV is a direct consequence of APOBEC3-mediated genome editing in the human host cannot be discerned at this stage. Also, the putative APOBEC3 effect on MPXV evolution augments the uncertainty regarding the 2022 outbreak origins and introductions, in addition to the complexity of the epidemiological context. This raises the need for future studies focusing on the weight of APOBEC3 in MPXV diversification. In particular, functional studies assessing whether this mutational driver triggers MPXV adaptive evolution toward altered phenotypic features, such as enhanced transmissibility, are warranted.

Further phylogenomic analysis revealed the first signs of microevolution of this virus during human-to-human transmission. Among the 15 outbreak sequences analyzed here, we detected the emergence of 15 SNPs (eight non-synonymous, four synonymous, two intergenic and one stop gained) (Fig. 2 and Supplementary Table 4). Notably, all SNPs also follow the same mutational bias, including eight GA > AA (six non-synonymous and two synonymous) and seven TC > TT (two non-synonymous, two synonymous, one stop gained and two intergenic) nucleotide replacements. This further suggests a continuous action of APOBEC3 during MPXV evolution. Among the seven phylogenetic branches directly descendant from the most recent ancestor of the MPXV outbreak strain (Fig. 1), we identified a subcluster (supported by two SNPs) of two sequences (PT0005 and PT0008, each with an additional SNP) that also share a 913-bp frameshift deletion in a gene coding for an ankyrin/host range (MPXV-UK_P2-010). Although gene loss events are not unexpected for orthopoxviruses (for example, variola virus has most likely undergone reductive evolution^26^), these were previously observed in the context of endemic MPXV circulation in Central Africa, being hypothesized to correlate with human-to-human transmission^27^.

Our data reveal additional clues of ongoing viral evolution and potential human adaptation. Most emerging SNPs in sequences from Portugal were not 100% fixed in the viral population (frequencies 75–95%), supporting the existence of viral intra-patient population diversity. Further inspection of minor intra-patient single-nucleotide variants (iSNVs) in Illumina samples led to the validation of 11 non-synonymous minor iSNVs (across five samples), again most with the ‘APOBEC3 signature’ (Supplementary Table 5). Notably, among the targeted viral transcripts, we highlight a few encoded proteins that are known to interact with host immune system, such as an MHC class II antigen presentation inhibitor^28^, an IFN-alpha/beta receptor glycoprotein^29^ and IL-1/TLR signaling inhibitor^30^. These and other proteins (Supplementary Tables 3–5) found to be targeted during the 2022 outbreak MPXV divergence and microevolution might constitute priority targets for future functional studies aiming to assess their potential role in adaptation.

In summary, our genomic and phylogenomic data provide insights into the evolutionary trajectory of the 2022 MPXV outbreak strain and shed light on potential mechanisms and targets of human adaptation. The observed accelerated evolution of this human MPXV, potentially driven by the APOBEC3 action, suggests that viral genome sequencing might provide sufficient resolution to track the transmission dynamics and outbreak spread, which seemed to be challenging for a presumably slow-evolving double-stranded DNA virus. Together with the adopted strategy of real-time data sharing, this study may help guide novel outbreak control measures and subsequent research directions.

## Methods

This research complies with all relevant ethical regulations. The Portuguese NIH (INSA) is the national reference laboratory, being the Portuguese laboratory authorized by the General Directorate of Health (through technical orientation no. 004/2022 of 31 May 2022) to process the samples for identification and characterization of MPXV. All samples subjected to viral genetic characterization are processed in an anonymized fashion.

### DNA extraction and shotgun metagenomics sequencing

All clinical samples (lesions and vesicle swabs) were received by the Emergency Response and Biopreparedness Unit at INSA and screened for MPXV by real-time polymerase chain reaction (PCR) targeting the *rpo18* gene^31^, on a CFX Opus Real-Time PCR System (Bio-Rad). The first set of samples (*n* = 2, received 10–11 May 2022) were subjected to DNA extraction using the QIamp DNA Mini Kit (Qiagen) before library preparation using the Rapid Barcoding Sequencing Kit (SQK-RBK004) and shotgun metagenomics sequencing on an Oxford Nanopore Technologies (ONT) MinION apparatus for 18 hours. We obtained the first draft genome sequence (Monkeypox/PT0001/2022) covering ~92% of the reference sequence, with a mean depth of coverage of seven-fold throughout the genome.

For the second set of samples (*n* = 13, received 16 May 2022), before DNA extraction a pre-treatment was performed by sonication (S30 Elmasonic) in a bath for 20 seconds, twice, followed by a DNase/RNase (1:10 solution of 18.5 mg of DNAse (Sigma-Aldrich) 400 Kunitz mg^−1^ + 52.14 mg of RNAse AppliChem 100.8 Kunitz mg^−1^ in HBSS 1×) treatment (20 minutes at 37 °C, 15 minutes at 65 °C and 1 minute on ice), to deplete host DNA. The DNA samples were then subjected to Nextera XT library preparation and subsequent shotgun metagenomics by paired-end sequencing (2 × 150 bp) on an Illumina NextSeq 2000 apparatus, with ~80 million total reads per sample. Mean depth of coverage throughout the monkeypox genome ranged from 38× to 508× (mean 201×). Seven out of the 13 samples were also subjected to ONT MinION sequencing, as previously described. Of note, although DNase/RNase treatment has been shown to perform well, results with the present sample set showed that we experienced wet lab technical issues affecting host depletion efficiency. Samples details are presented in Supplementary Table 1.

### Genome assembly

Reads were human-depleted using BMTagger^32^ and subsequently mapped to the reference genome MPXV-UK_P2 (MT903344.1, also being used as reference in the newest monkeypox nextstrain build: https://nextstrain.org/monkeypox) using the INSaFLU pipeline^33^ (https://insaflu.insa.pt/). In brief, Illumina reads were quality processed using FastQC version 0.11.5 and Trimmomatic version 0.27 and mapped using Snippy version 3.2, and ONT reads were quality processed using NanoStat version 1.4.0 and NanoFilt version 2.6.0 and mapped using medaka version 1.2.1. All genome sequences were further compared to de novo assemblies obtained using SPAdes version 3.11.1 for Illumina (to investigate the presence of large insertions/deletions or rearrangements), and all detected mutations were carefully inspected using Integrative Genomics Viewer software. Particularly, to characterize the mutational profile of the large inverted terminal repeats, Illumina reads were independently mapped against each of the terminal repeats of MPXV-UK_P2. As this confirmed that both terminal repeat regions were identical within each genome, these regions were manually joined to each end of the final genome sequences.

### Phylogenetics

A draft phylogenetic analysis for clade positioning (Fig. 1a) was conducted upon core SNP alignment (1,057 variant positions) retrieved from a rapid alignment (using parsnp version 1.2) of the newly sequenced genomes with publicly available genomes (Supplementary Table 2), with reference genome Zaire-96-I-16 (RefSeq accession no. NC_003310.1) set as an outgroup (clade 1). Fine-tune phylogeny of the 2022 outbreak-related genomes (Fig. 1b) was performed by aligning novel genomes (*n* = 15; Supplementary Table 2) with mafft version 7.487, followed by manual alignment curation and maximum likelihood phylogenetic tree construction using MEGA version 10 software. Of note, as one of the genomes (MPXV-CH-38134631/2022, ON595760.1) presented an excess of mutations in its terminal regions (known to be error-prone during sequencing), its positions were masked for phylogenetic analysis. The updated outbreak 2022 phylogenetic tree with sequences available in NCBI as of 15 June 2022 (Fig. 1c) was built similarly, with further masking (both outbreak alignments are available as Supplementary Data). Phylogenetic data visualization was performed with Microreact (https://microreact.org/).

### Microevolution analysis

Rapid extraction and/or visualization of variant sites from sequence alignments was performed using ReporTree (https://github.com/insapathogenomics/ReporTree), snipit (https://github.com/aineniamh/snipit) and NextClade (https://clades.nextstrain.org/). A Python script (https://github.com/insapathogenomics/mutation_profile) was developed to rapidly obtain the sequence context flanking all detected SNPs (including the outbreak cluster-defining mutations, ‘intra-cluster’ mutations and intra-host minor variants, described in Supplementary Tables 3–5, respectively) and screen whether they follow signatures potentially compatible with APOBEC3-mediated viral genome editing (namely, GA > AA and TC > TT replacements). Analysis of minor iSNVs displaying intra-sample frequency between 1% and 50% was performed using the pipeline implemented in INSaFLU (min-alternate-count set to 10), with the minimum ‘allele’ frequency being contingent on the depth of coverage of each processed site—that is, the identification of iSNV sites at frequencies of 10%, 2% and 1% is only allowed for sites with depth of coverage of at least 100-fold, 500-fold and 1,000-fold, respectively. Non-synonymous iSNVs were inspected in Integrative Genomics Viewer before validation (https://insaflu.insa.pt/)^33^.

### Statistical analyses

No specific advanced statistical methods were required for the data analysis of the present study.

### Reporting summary

Further information on research design is available in the Nature Research Reporting Summary linked to this article.

## Online content

Any methods, additional references, Nature Research reporting summaries, source data, extended data, supplementary information, acknowledgements, peer review information; details of author contributions and competing interests; and statements of data and code availability are available at 10.1038/s41591-022-01907-y.

## Supplementary information

Reporting Summary

Supplementary Data 1 Supplementary Table 1: Details of the samples subjected to shotgun metagenomics. Supplementary Table 2: Public MPXV genomes used for phylogenetic analysis. Supplementary Table 3: SNPs separating the monkeypox 2022 outbreak cluster from the reference genome sequence MPXV-UK_P2 (accession no. MT903344.1). Supplementary Table 4: Genetic diversity (SNPs) within the monkeypox 2022 outbreak virus (position refer to MPXV-UK_P2; accession no. MT903344.1). Supplementary Table 5: Non-synonymous minor iSNVs found in Illumina data from Portugal (position refer to MPXV_UK_P2; accession no. MT903344.1)

Supplementary Data 2 Alignments used to generate phylogenetic trees in Figure 1

## Data Availability

Monkeypox reads mapping to the reference sequence MPXV-UK_P2 (GenBank accession no. MT903344.1) were deposited in the European Nucleotide Archive (BioProject accession no. PRJEB53055). Assembled consensus sequences were deposited in the National Center for Biotechnology Information under accession nos. ON585029–ON585038. All accession numbers are included in Supplementary Table 1.

## Code availability

A Python script (https://github.com/insapathogenomics/mutation_profile) was developed to rapidly obtain the sequence context flanking all detected SNPs and screen whether they follow signatures potentially compatible with APOBEC3-mediated viral genome editing.
