# Addendum: Phylogenomic characterization and signs of microevolution in the 2022 multi-country outbreak of monkeypox virus

**DOI:** 10.1038/s41591-022-02036-2

**Published:** 2022-09-21

**Authors:** Joana Isidro, Vítor Borges, Miguel Pinto, Daniel Sobral, João Dourado Santos, Alexandra Nunes, Verónica Mixão, Rita Ferreira, Daniela Santos, Silvia Duarte, Luís Vieira, Maria José Borrego, Sofia Núncio, Isabel Lopes de Carvalho, Ana Pelerito, Rita Cordeiro, João Paulo Gomes

**Affiliations:** 1 Genomics and Bioinformatics Unit, Department of Infectious Diseases, National Institute of Health Doutor Ricardo Jorge (INSA), Lisbon, Portugal; 2 Technology and Innovation Unit, Department of Human Genetics, National Institute of Health Doutor Ricardo Jorge (INSA), Lisbon, Portugal; 3 National Reference Laboratory of Sexually Transmitted Infections, Department of Infectious Diseases, National Institute of Health Doutor Ricardo Jorge (INSA), Lisbon, Portugal; 4 Emergency Response and Biopreparedness Unit, Department of Infectious Diseases, National Institute of Health Doutor Ricardo Jorge (INSA), Lisbon, Portugal; 5 Faculty of Veterinary Medicine, Lusófona University, Lisbon, Portugal

**Keywords:** Viral infection, Viral genetics

Addendum to: *Nature Medicine* 10.1038/s41591-022-01907-y, published online 24 June 2022.

On 10 June 2022, a new nomenclature system for the monkeypox virus (MPXV) clades and lineages was proposed by Happi et al.^1^ that defined the Clade 1 (former Congo Basin) and Clades 2 and 3 (both comprising the former West African clade). Our study, published online in *Nature Medicine* on 24 June 2022, relied on this nomenclature. Later, on 12 August 2022, the World Health Organization, together with a group of global experts, revised and agreed on the new naming convention for MPXV clades in which the previously proposed Clades 1, 2 and 3 are now designated Clades I, IIa and IIb, respectively^2^. The initial proposal by Happi et al. was subsequently updated^1^ as well as the final corresponding publication^3^. In this context, the published version of our article (which relied on such proposal) should be read according to this official revision. In this regard, in the main text, supplementary material and figure captions, where it reads Clades 1, 2 and 3, it should read Clades I, IIa and IIb. A corrected version of Fig. 1 is also shown in this Addendum.

**Fig. 1:**
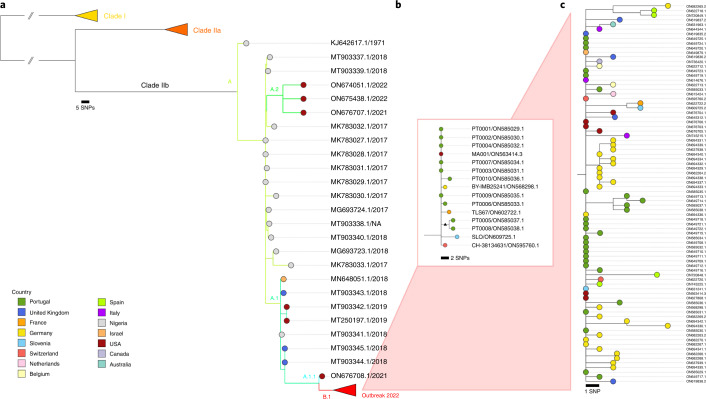
Phylogenetic analysis of MPXV viral sequences associated with the 2022 worldwide outbreak. **a**, MPXV global phylogeny showing that the 2022 outbreak cluster (lineage B.1) belongs to clade IIb. Clade and lineage are designated according to the nomenclature proposed by Happi et al.^3^. **b**, Genetic diversity within the outbreak cluster, including the 15 sequences analyzed in this study (released in the NCBI before 27 May 2022). The deletion symbol (Δ) denotes a large deletion (11,335–12,247 in the MPXV-UK_P2-010 gene) shared by sequences segregating in a small subcluster. **c**, Outbreak phylogenetic tree updated with sequences available in the NCBI as of 15 June 2022 (provided during revision for more updated contextualization). The list of the sequences used in these phylogenetic analyses is detailed in Supplementary Table 2, and the alignments are provided as Supplementary Data.
