# Supplementary material for: Phylogenomic characterization and signs of microevolution in the 2022 multi-country outbreak of monkeypox virus
Source: Nat Med. 2022 Jun 24;28(8):1569–72. doi: 10.1038/s41591-022-01907-y (PMC9388373; doi:10.1038/s41591-022-01907-y)
Supplement: Supplementary file 1 — Reporting Summary [file 41591_2022_1907_MOESM1_ESM.pdf]

nature research

Corresponding author(s): João Paulo Gomes

Last updated by author(s): Jun 18, 2022

Reporting Summary

Nature Research wishes to improve the reproducibility of the work that we publish. This form provides structure for consistency and transparency in reporting. For further information on Nature Research policies, see our Editorial Policies and the Editorial Policy Checklist.

Statistics

For all statistical analyses, confirm that the following items are present in the figure legend, table legend, main text, or Methods section.

n/a	Confirmed	
☐	☒	The exact sample size (*n*) for each experimental group/condition, given as a discrete number and unit of measurement
☒	☐	A statement on whether measurements were taken from distinct samples or whether the same sample was measured repeatedly
☒	☐	The statistical test(s) used AND whether they are one- or two-sided<br>*Only common tests should be described solely by name; describe more complex techniques in the Methods section.*
☒	☐	A description of all covariates tested
☒	☐	A description of any assumptions or corrections, such as tests of normality and adjustment for multiple comparisons
☒	☐	A full description of the statistical parameters including central tendency (e.g. means) or other basic estimates (e.g. regression coefficient) AND variation (e.g. standard deviation) or associated estimates of uncertainty (e.g. confidence intervals)
☒	☐	For null hypothesis testing, the test statistic (e.g. *F*, *t*, *r*) with confidence intervals, effect sizes, degrees of freedom and *P* value noted<br>*Give P values as exact values whenever suitable.*
☒	☐	For Bayesian analysis, information on the choice of priors and Markov chain Monte Carlo settings
☒	☐	For hierarchical and complex designs, identification of the appropriate level for tests and full reporting of outcomes
☒	☐	Estimates of effect sizes (e.g. Cohen's *d*, Pearson's *r*), indicating how they were calculated

Our web collection on statistics for biologists contains articles on many of the points above.

Software and code

Policy information about availability of computer code

Data collection	Not applicable
Data analysis	INSaFLU v.1.5.2 (https://insaflu.insa.pt/; code: https://github.com/INSaFLU/INSaFLU), including FastQC v0.11.5 (https://www.bioinformatics.babraham.ac.uk/projects/fastqc/), Trimmomatic v0.27 (http://www.usadellab.org/cms/index.php?page=trimmomatic), NanoStat v1.4.0 (https://github.com/wdecoster/nanostat), NanoFilt v.2.6.0 (https://github.com/wdecoster/nanofilt), RabbitQC 0.0.1 (https://github.com/ZekunYin/RabbitQC), SPAdes v3.11.1 (http://cab.spbu.ru/software/spades/), Snippy v.3.2-dev (https://github.com/tseemann/snippy, sligthly modified in https://github.com/INSaFLU/INSaFLU), Medaka v1.2.1 (https://nanoporetech.github.io/medaka/), msa_masker (https://github.com/rfm-targa/BioinfUtils/blob/master/msa_masker.py), Integrative Genomics Viewer (http://software.broadinstitute.org/software/igv/), getCoveragev1.1 (https://github.com/monsanto-pinheiro/getCoverage), ReporTree (https://github.com/insapathogenomics/ReporTree); get_mutation_profile (https://github.com/insapathogenomics/mutation_profile); MAFFT v7.487 (https://mafft.cbrc.jp/alignment/software/); MEGA v10 (https://www.megasoftware.net/); snipit (https://github.com/aineniamh/snipit), Microreact (https://microreact.org/), Nextclade 2.1.0 (https://clades.nextstrain.org/).

For manuscripts utilizing custom algorithms or software that are central to the research but not yet described in published literature, software must be made available to editors and reviewers. We strongly encourage code deposition in a community repository (e.g. GitHub). See the Nature Research guidelines for submitting code & software for further information.

Data

Policy information about availability of data

All manuscripts must include a data availability statement. This statement should provide the following information, where applicable:

- Accession codes, unique identifiers, or web links for publicly available datasets
- A list of figures that have associated raw data
- A description of any restrictions on data availability

Monkeypox reads mapping to the reference sequence MPXV-UK_P2 (MT903344.1) were deposited in the European Nucleotide Archive (ENA) (BioProject accession no. PRJEB53055; www.ebi.ac.uk/ena/data/view/PRJEB53055). Reads maping against the human genome were removed before submission. Assembled consensus sequences were deposited in the National Center for Biotechnology Information (NCBI) under the accession numbers ON585029-ON585038. All accession numbers are included in Supplementary Table 1.

Field-specific reporting

Please select the one below that is the best fit for your research. If you are not sure, read the appropriate sections before making your selection.

☒ Life sciences ☐ Behavioural & social sciences ☐ Ecological, evolutionary & environmental sciences

For a reference copy of the document with all sections, see nature.com/documents/nr-reporting-summary-flat.pdf

Life sciences study design

All studies must disclose on these points even when the disclosure is negative.

Sample size	To define the sample size we opted for sequencing the viral genomes from all the samples that were available at the Ref Lab at the time we performed the wet-lab procedures. The goal was to provide a first robust snapshot on phylogenomics of the 2022 Monkeypox virus.
Data exclusions	Samples presenting a very low viral load (defined by the real-time PCR threshold cycle values) were excluded.
Replication	Not applicable. Shotgun metagenomics sequencing was performed for each specimen, with robustness of sequence data being ensured by the applied depth of coverage and data analysis.
Randomization	As described above, all available suitable samples were processed at that time.
Blinding	Blinding was not relevant/applicable. We analyzed all available samples strictly for genomic purposes and the data is anonymized.

Reporting for specific materials, systems and methods

We require information from authors about some types of materials, experimental systems and methods used in many studies. Here, indicate whether each material, system or method listed is relevant to your study. If you are not sure if a list item applies to your research, read the appropriate section before selecting a response.

Materials & experimental systems

n/a	Involved in the study	
☒	☐	Antibodies
☒	☐	Eukaryotic cell lines
☒	☐	Palaeontology and archaeology
☒	☐	Animals and other organisms
☒	☐	Human research participants
☒	☐	Clinical data
☒	☐	Dual use research of concern

Methods

n/a	Involved in the study	
☒	☐	ChIP-seq
☒	☐	Flow cytometry
☒	☐	MRI-based neuroimaging
